# Dietary choline, but not L-carnitine, increases circulating lipid and lipoprotein concentrations, without affecting body composition, energy expenditure or respiratory quotient in lean and obese male cats during weight maintenance

**DOI:** 10.3389/fvets.2023.1198175

**Published:** 2023-07-26

**Authors:** Alexandra Rankovic, Shoshana Verton-Shaw, Anna K. Shoveller, Marica Bakovic, Gordon Kirby, Adronie Verbrugghe

**Affiliations:** ^1^Department of Biomedical Sciences, Ontario Veterinary College, University of Guelph, Guelph, ON, Canada; ^2^Department of Clinical Studies, Ontario Veterinary College, University of Guelph, Guelph, ON, Canada; ^3^Department of Animal Biosciences, Ontario Agricultural College, University of Guelph, Guelph, ON, Canada; ^4^Department of Human Health and Nutritional Sciences, College of Biological Sciences, University of Guelph, Guelph, ON, Canada

**Keywords:** dual-energy x-ray absorptiometry, feline nutrition, indirect calorimetry, methionine, methyl donor, one carbon metabolism, pet obesity

## Abstract

**Introduction:**

Due to the involvement in one-carbon metabolism and lipid mobilization, choline and L-carnitine supplementation have been recommended to minimize hepatic lipid accumulation and support fat oxidation, respectively. This study investigated the lipotropic benefits of choline or L-carnitine supplementation in lean and obese cats maintaining body weight (BW).

**Methods:**

Lean [*n* = 9; body condition score (BCS): 4–5/9] and obese (*n* = 9; BCS: 8–9/9) adult male neutered colony cats were used in a replicated 3 x 3 complete Latin square design. Treatments included choline (378 mg/kg BW^0.67^), L-carnitine (200 mg/kg BW) and control (no supplement). Treatments were supplemented to the food for 6 weeks each, with a 2-week washout between treatments. Cats were fed once daily to maintenance energy requirements, and BW and BCS were assessed weekly. Fasted blood collection, indirect calorimetry, and dual-energy X-ray absorptiometry occurred at the end of each treatment period. Serum was analyzed for cholesterol (CHOL), high-density lipoprotein CHOL (HDL-C), triglycerides (TAG), non-esterified fatty acids (NEFA), glucose, creatinine (CREAT), urea, alkaline phosphatase (ALP) and alanine aminotransferase (ALT). Very low-density lipoprotein CHOL (VLDL) and low-density lipoprotein CHOL (LDL-C) were calculated. Data were analyzed using proc GLIMMIX, with group and period as random effects, and treatment, body condition, and their interaction as fixed effects, followed by a Tukey's *post-hoc* test when significance occurred.

**Results:**

Cats supplemented choline had lower food intake (*P* = 0.025). Treatment did not change BW, BCS and body composition (*P* > 0.05). Obese cats had greater ALP, TAG, and VLDL, and lower HDL-C compared to lean cats (*P* < 0.05). Choline resulted in greater CHOL, HDL-C, LDL-C and ALT (*P* < 0.05). L-carnitine resulted in lower CREAT (*P* = 0.010). Following the *post-hoc* test, differences between treatment means were not present for ALP (*P* = 0.042). No differences were found for glucose, urea or NEFA (*P* > 0.05). Obese cats had a lower fed respiratory quotient (RQ), regardless of treatment (*P* = 0.045). Treatment did not affect fed or fasted RQ and energy expenditure (*P* > 0.05).

**Discussion:**

Choline appeared to increase circulating lipid and lipoprotein concentrations regardless of body condition, likely through enhanced lipid mobilization and hepatic elimination. Neither dietary choline or L-carnitine altered body composition or energy metabolism in the lean or obese cats, as compared to control.

## 1. Introduction

Feline hepatic lipidosis (FHL) is defined by the excess storage of hepatic lipids and is considered the most common liver disease affecting cats in North America (1). Although the exact pathophysiology of FHL has not been identified, there is evidence that the mobilization of free fatty acids from the adipose tissue leads to the accumulation of lipids within the livers of affected cats (2). Food deprivation, whether due to secondary anorexia or due to an imposed high degree of energy restriction, is considered a key factor in the development of FHL (1, 3–6). Left untreated, the prognosis for affected cats is considered poor and may result in liver failure and death (7). Obese cats are at an increased risk of developing FHL due to their increased levels of adipose tissue and subsequent stores of fatty acids available for mobilization (2, 8). Also, higher concentrations of hepatic triglycerides (TAG) and an increased risk of concurrent insulin resistance are believed to leave obese cats more susceptible to FHL (9, 10). Additionally, energy restriction is often recommended in obese cats in order to lose weight. However, a low degree of dietary energy restriction often does not result in weight loss in an obese cat, and therefore may result in a higher degree of energy restriction may be implemented (11, 12).

Under normal conditions, fatty acids within the hepatocytes can undergo β-oxidation within the mitochondria for the production of acetyl-CoA, or they can be re-esterified to TAG (13, 14). These TAG can be secreted into the circulation or they can continue to accumulate within the hepatocytes. It is unclear what leads to the accumulation of hepatic TAG during FHL. However, the metabolic idiosyncrasies that result from their obligate carnivorous nature put cats at risk for deficiencies in certain dietary essential nutrients. Insufficient intakes of dietary choline and carnitine during energy restriction have been proposed as potential risk factors in the pathogenesis of FHL (15).

Choline is a precursor for the biosynthesis of phosphatidylcholine (PC). Specifically, PC is required for packaging TAG and cholesterol into very low-density lipoproteins (VLDL) for export out of the liver (16). Betaine, a derivative of choline, also serves as a methyl group donor for one of the pathways through which homocysteine is remethylated to methionine. The production of methionine subsequently results in the methylation and synthesis of numerous metabolites, including carnitine (17). Previous research in mammals, including cats, found that choline deficiency leads to the development of fatty liver, which can be reversed and/or prevented when dietary choline is added back to the diet in sufficient quantities (18–20). In cats, increased dietary choline supplementation leads to increased concentrations of serum lipids and lipoproteins in both obese and overweight cats (21, 22), suggesting increased lipid hepatic mobilization through the synthesis and secretion of VLDL from the liver. Additionally, choline and its derivative betaine may reduce fat mass gain, as suggested in livestock species and growing kittens fed *ad libitum* post-gonadectomy (23–25).

In comparison, carnitine is required to facilitate the entry of fatty acids into the mitochondria for β-oxidation. This is considered the main pathway for the disposal of fatty acids under normal physiological conditions and is important for the production of ATP by the mitochondria (13). L-carnitine increases lipid oxidation in overweight cats, as suggested by increased palmitate oxidation (26), energy expenditure (EE) and lower post-prandial respiratory quotient (RQ) (27). Additionally, L-carnitine supplementation may decrease food intake and weight gain in *ad libitum* feeding scenarios (28). For these reasons, L-carnitine is commonly used by the pet food industry in the formulation of weight control and weight loss diets for cats. In human medicine, reduced β-oxidation has been suggested as a potential cause of lipid accumulation in the liver of patients with non-alcoholic fatty liver disease (NAFLD) (29). Thus, it has been proposed that an insufficient supply of carnitine may similarly be an important factor for the development of FHL, although the evidence to support this has been inconsistent (30–34).

Nutrients and nutraceuticals that can reduce the risk of hepatic lipid accumulation in obese cats and assist in mitigating weight gain would benefit the pet food industry and the veterinary community. Currently, there is evidence that dietary choline may provide such benefits to overweight and obese cats (21, 22). However, there have been no studies comparing the lipotropic benefits of dietary choline to L-carnitine, a dietary supplement commonly used by the pet food industry. Therefore, the objective of this research was to investigate the lipotropic effects of dietary choline compared to L-carnitine (a positive control) and no supplement (a negative control), on serum lipid and lipoprotein profiles, body composition, EE and RQ in obese and lean adult cats. We hypothesized that supplemental choline would result in greater serum lipid and lipoprotein concentrations, as compared to L-carnitine or control, whereas L-carnitine would increase EE and lower RQ only in the obese cats, suggesting favored fatty acid oxidation. Additionally, we hypothesized that both choline and L-carnitine would improve body composition by decreasing fat mass and increasing lean body mass only in the obese cats, as compared to control.

## 2. Materials and methods

All procedures were approved by the University of Guelph Animal Care Committee (AUP#4496), in compliance with provincial and national guidelines regarding the care and use of animals in research.

### 2.1. Animals and housing

Eighteen domestic shorthair (DSH) male neutered cats (Marshall's Bio Resources, Waverly, NY, United States of America) were enrolled in this trial. The cats were between 1 to 2 years of age (mean ± SEM: 2.00 ± 0.11 years; range: 1.28–2.29 years) and were considered healthy prior to the start of the trial, based on physical examination, medical history, and the results of complete blood count (CBC) and serum biochemistry completed within 6 months of the trial. The cats in the present study were fed using a modified *ad libitum* protocol during growth (25). At the start of the trial, the 18 cats were classified into two groups, “obese” or “lean”, based on assigned body condition score (BCS) (35). Nine of the 18 cats were classified as obese, with a BCS of ≥ 8/9 (36), and a mean body weight (BW) of 6.46 ± 0.15 kg. The remaining nine were classified as lean, with an assigned BCS of 4–5/9 and BW of 4.62 ± 0.15 kg.

All 18 cats were housed together in a free-living environment (23 ft x 19 ft) at the Animal Biosciences Cattery at the Ontario Agricultural College of the University of Guelph (Guelph, ON, Canada). The room had a controlled 12 h light 12 h dark cycle, with the lights turning on at 0700 h and off at 1,900 h. Humidity and temperature were maintained at 40% and 24°C respectively throughout the trial.

Water was available to the cats *ad libitum* in bowls and through an open tap. The room was cleaned daily and enrichment was provided through regular interaction with familiar people and within the environment, as previously described in Frayne et al. (37).

### 2.2. Diet

Four weeks leading up to the trial (adaptation period) and throughout the entire length of the trial, cats remained on a commercial extruded diet (Nutram Total Grain-Free® Chicken and Turkey Recipe, Elmira Pet Products, Elmira, ON, Canada) formulated for feline adult maintenance, per the Association of American Feed Control Officials (AAFCO) (35). To be fed individually, cats were separated and placed in individual cages for 1 h daily (08:00 h). Food was provided in quantities to maintain current BW based on historic dietary intake data. Individual orts were measured and recorded daily. Fasted BW and BCS were recorded weekly by the same assessor (A.R.).

As previously detailed in Rankovic et al. (22), nutrient analysis of the diet (Table 1) was performed using methods described by the Association of Official Analytical Chemists (AOAC) and the American Oil Chemist Society (AOCS) (Bureau Veritas, Mississauga, ON, Canada) (38, 39). This included the measurement of moisture (AOAC 935.29), crude protein (AOAC 990.03), crude fat (AOAC 920.39), crude fiber (AOCS Ba6a-05), ash (AOAC 942.05), total dietary fiber (AOAC 991.43, 985.29), pyridoxine [B6, AOAC 985.32 (modified)], folate (B9; AOAC 2004.5), cobalamin (B12; AOAC 986.23), choline (AOAC 999.14). Dietary carnitine concentrations were measured by liquid chromatography-mass spectrometry, according to AOAC 2012.17 (38). In addition, concentrations of dietary amino acids were measured using ultra-performance liquid chromatography (UPLC), as previously described by Cargo-Froom et al. (40). Nitrogen free extract (NFE) was calculated by difference (NFE % = 100-moisture-protein-fat-crude fiber-ash), and metabolizable energy (ME) was estimated using the Modified Atwater equation [ME = 10 x (3.5 x %crude protein) + (8.5 x %fat) + (3.5 x %NFE)] (41).

**Table 1:** The proximate analysis, energy content, fiber, choline, carnitine, selected B-vitamin and amino acid concentrations of a commercial extruded adult cat food fed at maintenance energy requirements to obese (*n* = 9) and lean adult cats (*n* = 9).

Moisture	% as-fed	4.3
Protein	% DM	41.7
Fat	% DM	17.5
Ash	% DM	8.8
Crude fiber	% DM	1.5
Total dietary fiber	% DM	4.5
NFE^b^	% DM	30.6
ME^a^	Kcal/kg	3,864.0
Choline	mg/100 g	428.4
Carnitine	mg/100 g	4.6
Cobalamin (B12)	mg/100 g	9.2
Pyridoxine (B6)	mg/100 g	1.4
Folate (B9)	mg/100 g	0.2
Alanine	% DM	2.4
Arginine	% DM	2.7
Aspartate	% DM	3.6
Cysteine	% DM	1.5
Glutamine	% DM	5.3
Glycine	% DM	3.4
Histidine	% DM	0.9
Isoleucine	% DM	1.6
Leucine	% DM	2.9
Lysine	% DM	2.5
Methionine	% DM	0.9
Phenylalanine	% DM	1.8
Proline	% DM	2.5
Serine	% DM	1.8
Taurine	% DM	0.2
Threonine	% DM	1.5
Tryptophan	% DM	0.2
Tyrosine	% DM	1.3
Valine	% DM	1.8

Reported on a dry matter basis (DMB) apart from moisture.

a Metabolizable Energy (ME) calculated using the Modified Atwater Equation: ME = 10 x [(3.5 x %crude protein) + (8.5 x %fat) + (3.5 x %NFE)] (41).

b Nitrogen Free Extract (NFE) % = 100–moisture–protein–fat-crude fiber-ash (41); Ingredients: Deboned Chicken, Deboned Turkey, Chicken Meal, Whole Eggs, Turkey Meal, Lentils, Peas, Chickpeas, Chicken Fat (preserved with Mixed Tocopherols), Split Peas, Flaxseed, Natural Chicken Flavor, Pumpkin, Broccoli, Quinoa Seed, Dried Cranberries, Choline Chloride, Pomegranate, Raspberries, Kale, Salt, Chicory Root Extract, Vitamins and Minerals (Vitamin E Supplement, Niacin (source of Vitamin B3), Vitamin A Supplement, Thiamine Mononitrate (source of Vitamin B1), d-Calcium Pantothenate (source of Vitamin B5), Pyridoxine Hydrochloride (source of Vitamin B6), Riboflavin (source of Vitamin B2), Beta-Carotene, Vitamin D3 Supplement, Folic Acid, Biotin, Vitamin B12 Supplement, Zinc Proteinate, Ferrous Sulfate, Zinc Oxide, Iron Proteinate, Copper Sulfate, Copper Proteinate, Manganese Proteinate, Manganous Oxide, Calcium Iodate, Sodium Selenite), DL-Methionine, Taurine, Yucca schidigera Extract, Spinach, Celery Seeds, Peppermint, Chamomile, Turmeric, Ginger, Rosemary.

### 2.3. Study design and dietary choline and L-carnitine supplementation

All cats received each of the three treatments (choline, carnitine and control) in a 3 x 3 Latin square design. Prior to the trial, the 18 cats were split into three groups of six. Each group was balanced for BW and consisted of three obese and three lean cats. Each treatment was provided for a 6-week period, and cats were placed on a 2-week washout between periods where they received only the commercial cat food with no supplementation. The same food without supplementation continued to be provided in the same quantities to maintain BW during washout, as during the treatment periods.

As per previous research (22), choline (PuraChol, 70% aqueous choline chloride, 52.2% choline ion; Balchem Corporation, New Hampton, NY, United States of America) was supplemented to provide a daily choline intake of six times the recommended allowance (RA) for dietary choline published by the National Research Council (NRC) [6 x 63 mg/kg BW^0.67^ (378 mg/kg BW^0.67^)] (22, 41). The choline was measured daily, before feeding, with an adjustable volume pipette (Research plus™ Variable Adjustable Volume Pipette: 100–1,000 μL Single-Channel, Eppendorf Canada Ltd., Mississauga, ON, Canada). The estimated choline intake from the diet (4.3 mg choline/g diet as-fed) was accounted for when calculating the choline supplement dose for each individual cat. L-carnitine (48.5% L-carnitine tartrate, Bill Barr & Company, Overland Park, KS, United States of America) was supplemented at 200 mg/kg BW daily (26, 28). The L-carnitine was pre-measured daily before feeding and was mixed with a small amount of water. Each cat received their food once daily. Prior to feeding, each cat's daily food amount was split into two rations. The first ration provided $\frac{1}{4}$ of the daily food intake. The dietary treatments (choline or L-carnitine) were top-dressed onto this first ration and left to soak for 20 min prior to feeding. Once consumed, each cat was provided their second ration (3/4 daily food intake). Cats on the control treatment and during washout similarly received two rations but did not have anything top-dressed onto their first ration.

### 2.4. Indirect calorimetry

Indirect calorimetry was performed on the last day of each 6-week period (day 42), to determine individual EE and RQ. Each 24-h session comprised of a gas equilibrium period of 30 min, pre-prandial (fasted) measurements (1.5 h), and fed and extended postprandial measurements (22 h). Cats were acclimated to the chambers, using the methods previously outlined by Gooding et al. (42). Calibration of gases and protocol followed the methods previously described (22, 25).

Briefly, an open circuit, ventilated system (Qubit C950 Multi Channel Gas Exchange, Qubit Systems Inc., Kingston, ON, Canada) was used, with plexiglass chambers measuring 53 x 53 x 79 cm (length x width x height). The volume of space within the chamber measured 221.91 L. To maintain levels of CO_2_ within the chambers between 0.4% and 0.7%, room air was pulled through the chambers at a flow rate between 4.1–6.2 L/min.

The cats had access to a litter box, a water bowl and a hammock within the chamber. Water was filled before placing the cats in the calorimetry chambers and starting measurements. During each calorimetry session, cats continued to receive their food and treatment in the same 2-ration method as outlined previously.

Respiratory quotient and EE were calculated by the C950-Multi Channel Gas Exchange system (Qubit Systems Inc, Kingston, ON, Canada), using the following equations (43):


$$\begin{array}{l} Respiratory\;Quotient\;\left(RQ\right)\;=\;CO2\;produced\;\left(L\right)\;/\;O2\;consumed\;\left(L\right) \end{array}$$

$$\begin{array}{l} Energy\;Expenditure\;\left(EE\right)\;\left(kcal\right)\;=\;3.94\;\times \;O2\;consumed\;\left(L\right)\\ +\;1.11\;\times \;CO2\;produced\;\left(L\right) \end{array}$$

### 2.5. Blood collection and laboratory analyses

Following the completion of each 24 h indirect calorimetry session, cats were anesthetized for blood and tissue sample collection. Tissue samples are subject to further analysis and are not part of this study. Cats were pre-medicated with hydromorphone (0.05 mg/kg BW IM) and acepromazine (0.04 mg/kg BW IM), and induced with alfaxalone (1–3 mg/kg BW IV, to effect) and midazolam (0.3 mg/kg BW IV) (44). Fasted blood samples were collected from the jugular vein (5 mL). Following collection, the whole blood was transferred to serum-separating tubes and stored at 5°C until centrifugation. Centrifugation of collected whole blood occurred within 10 h of collection. Samples were centrifuged at 2,500 g x 15 min at 4°C (LegendRT, Kendro Laboratory Products 2002, Germany). Serum was separated, aliquoted and submitted (0.5 mL) to the Animal Health Laboratory at the University of Guelph (Guelph, ON, Canada) for analysis of serum CHOL, TAG, non-esterified fatty acids (NEFA), high-density lipoprotein CHOL (HDL-C), alkaline phosphatase (ALP), alanine aminotransferase (ALT), blood urea nitrogen (BUN), creatinine (CREAT) and glucose (GLUC), via photometry using a Roche Cobas 6000 c501 analyzer (Roche Diagnostics, Basel, Switzerland). The Friedewald equation was used to calculate very low-density lipoprotein CHOL (VLDL) and low-density lipoprotein CHOL (LDL-C) (45):


$$\begin{array}{l} Very\;low-density\;lipoprotein\;CHOL\;\left(VLDL\right)\;\left(mmol/L\right)\\ =\;TAG\;\left(mmol/L\right)\;/\;2.2 \end{array}$$

$$\begin{array}{l} Low-density\;lipoprotein\;CHOL\;\left(LDL-C\right)\;\left(mmol/L\right)\\ =\;total\;CHOL\;\left(mmol/L\right)\;-\;HDL-C\;\left(mmol/L\right)\\ -\;VLDL\;\left(mmol/L\right) \end{array}$$

### 2.6. Dual energy x-ray absorptiometry

Following blood collection, on the same day, body composition was assessed by dual energy x-ray absorptiometry (DXA). If needed, cats were administered additional sedation using dexmedetomidine (0.3 mg/kg BW IM). Atipamezole (0.2 mg/kg BW IM) was used to reverse sedation following DXA scans (44). Scans were performed in duplicate for each individual cat using a fan-beam DXA device (Prodigy® Advance GE Healthcare, Madison, WI, United States of America) to estimate whole body fat % (BF %), total tissue mass (TTM), fat mass (FM), and lean soft tissue mass (LSTM) (enCORE Version 16; GE Healthcare, Madison, WI, United States of America). The Small Animal Mode with Thin Setting was used. Each cat was individually positioned onto the DXA scanner in dorsal recumbency with forelimbs extended cranially (46). The cats were repositioned as necessary between scans. Each scan lasted ~10 min.

### 2.7. Statistical analyses

Residuals of serum biochemistry and lipoprotein profile, body composition, indirect calorimetry, BW, BCS, and intake data were all assessed for normality with the Shapiro-Wilk test. Food intake, energy intake, BCS, fed EE, fed RQ, choline and carnitine intake (mg/day, mg/kg BW, and mg/kg BW^0.67^) were not normally distributed and underwent a log transformation as a result. The data was back-transformed to obtain least square means (LSM) for each response variable.

The trapezoidal method was used to calculate area under the curve for RQ (AUC_RQ_), both in the fasted (pre-prandial) and fed states (0–120 min, 120–480 min, 480–820 min, 820–1,300 min) (9.3.1, GraphPad Software, San Diego, CA, United States of America). Statistical analyses of serum ALP, ALT, BUN, CREAT, GLUC, lipid and lipoprotein concentrations (CHOL, NEFA, HDL-C, LDL-C, VLDL, and TAG), body composition data (BF %, TTM, FM, and LSTM), EE, RQ and AUC_RQ_, were performed using SAS (SAS Studio 3.8, SAS Institute, Cary, NC, United States) as a generalized linear mixed model. The proc GLIMMIX procedure was used, with treatment, “body condition” (obese or lean), and treatment x body condition interaction set as the fixed effects, period and group as the random effects, and cat as the subject. Food intake and BW were included as covariates when assessing differences in RQ and EE. The covariance matrix resulting in the smallest Akaike information criterion value was used.

Differences in BW and BCS over time were similarly assessed through Proc GLIMMIX with treatment, body condition and their subsequent interaction as fixed effects, period and group as the random effects, week as the repeated term, and cat as the subject. Baseline BCS and BW were used as covariates when assessing change in BW and BCS.

Tukey's *post hoc* test was performed for all multiple comparisons where a significant effect of treatment, body condition, or treatment and body condition interaction was present. Results are expressed as LSM ± standard error of mean (SEM). A *P*-value < 0.05 was considered significant, and a *P*-value of < 0.10 was considered a trend.

## 3. Results

One obese cat was removed during period three due to a medical condition not related to the study. As a result, data from this cat were not included for period three (choline treatment). Supplements were accepted by the cats with no observed adverse health effects throughout the trial.

### 3.1. Food, energy, choline, and L-carnitine intake

Obese cats had greater food intake and subsequent energy intake compared to lean cats (P_Condition_ < 0.001; Table 2). Choline, but not L-carnitine, resulted in lower food and energy intake in both lean and obese cats (P_Treatment_ = 0.025), with no effect of treatment x body condition interaction (P_TreatmentxCondition_= 0.669).

**Table 2:** Food, energy, choline and L-carnitine intake of lean (*n* = 9) and obese (*n* = 9 for control and L-carnitine; *n* = 8 for choline) adult cats receiving supplemental dietary choline (378 mg/kg BW^0.67^), supplemental L-carnitine (200 mg/kg BW), or control (no additional supplement) top-dressed onto a commercial extruded adult feline diet and fed to maintenance energy requirements in a 3 x 3 Latin square design for 6-week periods.

	**Body condition (BC)**	**Treatment (T)**			***P*** **-values**		
		**Control (*****n** =* **18)**	**Choline (*****n** =* **17)**	**L-carnitine (*****n** =* **18)**	**BC**	**T**	**BC X T**
Food intake (G/Day)	Lean	53.84 ± 1.69	52.63 ± 1.65	52.21 ± 1.6	**<0.001**	**0.025**	0.669
	Obese	67.82 ± 2.13	65.27 ± 2.05	66.15 ± 2.08			
Energy intake (kcal/day)	Lean	207.03 ± 6.51	202.39 ± 6.36	200.76 ± 6.3	**<0.001**	**0.025**	0.669
	Obese	260.80 ± 8.20	250.97 ± 7.90	254.39 ± 8.00			
Choline intake (mg/day)	Lean	220.55 ± 5.46	1,049.38 ± 25.57	213.63 ± 5.2	**<0.001**	**<0.001**	0.696
	Obese	277.97 ± 6.89	1,300.90 ± 32.29	271.31± 6.73			
Choline intake (mg/kg BW)	Lean	48.18 ± 0.90	227.24 ± 4.20	46.76 ± 0.8	**<0.001**	**<0.001**	0.810
	Obese	43.61 ± 0.82	202.52 ± 3.80	42.44 ± 0.79			
Choline intake (mg/kg BW^0.67^)	Lean	79.63 ± 1.41	376.57 ± 6.59	77.22 ± 1.3	0.770	**<0.001**	0.770
	Obese	80.36 ± 1.42	374.13 ± 6.62	78.28 ± 1.38			
Carnitine intake (mg/day)	Lean	2.48 ± 0.08^Ad^	2.42 ± 0.07^Ad^	917.46 ± 27.76^A^	**<0.001**	**<0.001**	**<0.001**
	Obese	3.12 ± 0.10^Bd^	3.00 ± 0.09^Bd^	1,282.28 ± 39.31^Bc^			
Carnitine intake (mg/kg BW)	Lean	0.54 ± 0.01^Ad^	0.52 ± 0.01^Ad^	200.53 ± 4.41^A^	**0.003**	**<0.001**	**0.002**
	Obese	0.49 ± 0.01^Bd^	0.47 ± 0.01^Bd^	200.58 ± 4.42 ^Ac^			
Carnitine intake (mg/kg BW^0.67^)	Lean	0.89 ± 0.02^Ad^	0.87 ± 0.02^Ad^	331.13 ± 7.47^A^	0.127	**<0.001**	**0.001**
	Obese	0.90 ± 0.02^Ad^	0.86 ± 0.02^Ad^	369.95 ± 8.38^Bc^			

Values expressed as LSM ± SEM. Down a column, different upper case letter superscripts (A,B), represent significant difference between body conditions within a treatment, where a *p*-value of less than 0.05 is considered significant (represented in bold); Across a row, different lower case letter superscripts (c,d), represent significant difference between treatments within a body condition, where a *p*-value of less than 0.05 is considered significant; Repeated measures ANOVA with Tukey *post-hoc* test. BW, body weight; T, effect of treatment; BC X T, effect of body condition by treatment interaction.

As expected, treatment affected choline and L-carnitine intake (P_Treatment_ < 0.001), where supplementation resulted in greater intake. Additionally, there was an effect of body condition when choline and L-carnitine intake were expressed on a mg/day and mg/kg BW basis (Mg/day: P_Condition_ < 0.001, and < 0.001, respectively; mg/kg BW: P_Condition_ < 0.001, and 0.003, respectively). When expressed as mg/day, choline and L-carnitine intake were greater in obese cats. However, obese cats had a lower intake of both choline and L-carnitine when expressed on a mg/kg BW basis. Treatment x body condition interaction also affected L-carnitine intake (mg/day: P_TreatmentxCondition_ < 0.001; mg/kg BW: P_TreatmentxCondition_ = 0.002; mg/kg BW^0.67^: P_TreatmentxCondition_ = 0.001), where obese cats receiving the L-carnitine treatment had the greatest intakes of L-carnitine. There was no effect of treatment x body condition interaction on choline intake (mg/day: P_TreatmentxCondition_ 0.696; mg/kg BW: P_TreatmentxCondition_ = 0.810; mg/kg BW^0.67^: P_TreatmentxCondition_ = 0.770).

### 3.2. Body weight, body condition score and body composition

Body weight, BCS and body composition data are presented in Table 3. Both BW and BCS were higher in the obese cats than in the lean cats (P_Condition_ < 0.001) and neither changed treatment or treatment x body condition interaction (P_Treatment_ = 0.419, and 0.667, respectively; P_TreatmentxCondition_= 0.885, and 0.667 respectively). There were no changes in BW or BCS over time, in both lean and obese cats, or with any of the three treatments (*P* > 0.05; data not shown).

**Table 3:** Body weight, BCS, and body composition of lean (*n* = 9) and obese (*n* = 9 for control and L-carnitine; *n* = 8 for choline) adult cats receiving supplemental dietary choline (378 mg/kg BW^0.67^), supplemental L-carnitine (200 mg/kg BW), or control (no additional supplement top-dressed onto a commercial extruded adult feline diet) and fed to maintenance energy requirements in a 3 x 3 Latin square design for 6-week periods.

	**Body condition (BC)**	**Treatment (T)**			***P*** **-values**		
		**Control (*****n** =* **18)**	**Choline (*****n** =* **17)**	**L-carnitine (*****n** =* **18)**	**BC**	**T**	**BC X T**
BW (kg)	Lean	4.59 ± 0.03	4.63 ± 0.03	4.58 ± 0.0	**<0.001**	0.419	0.885
	Obese	6.39 ± 0.03	6.44 ± 0.03	6.41 ± 0.03			
BCS	Lean	4.88 ± 0.12	4.88 ± 0.12	4.88 ± 0.1	**<0.001**	0.667	0.667
	Obese	7.88 ± 0.20	8.04 ± 0.20	7.93 ± 0.20			
Total Tissue mass (g)	Lean	4,360.89 ± 142.84	4,420.00 ± 142.84	4,353.72 ± 142.8	**<0.001**	0.309	0.734
	Obese	6,081.83 ± 142.84	6,128.50 ± 142.84	6,114.78 ± 142.84			
% Body fat	Lean	18.17 ± 1.25	18.28 ± 1.25	17.37 ± 1.2	**<0.001**	0.525	0.384
	Obese	32.30 ± 1.25	31.80 ± 1.25	32.12 ± 1.25			
Fat mass (G)	Lean	795.44 ± 84.88	807.33 ± 84.88	760.11 ± 84.8	**<0.001**	0.847	0.569
	Obese	1,965.72 ± 84.88	1,949.60 ± 84.88	1,968.61 ± 84.88			
Lean soft tissue mass (g)	Lean	3,565.50 ± 110.13	3,612.50 ± 110.13	3,593.61 ± 110.1	**0.001**	0.066	0.858
	Obese	4,115.61 ± 110.13	4,187.18 ± 110.13	4,146.06 ± 110.13			

Values expressed as LSM ± SEM; *P* < 0.05 (represented in bold), Repeated measures ANOVA with Tukey *post-hoc* test. BW, body weight; BCS, body condition score; BC, effect of body condition; T, effect of treatment; BC X T, effect of body condition by treatment interaction.

As expected, TTM, FM, and BF % were higher in the obese cats than in the lean cats (P_Condition_ < 0.001). However, treatment and treatment x body condition interaction did not change any of these parameters (P_Treatment_ = 0.309, 0.847, and 0.525, respectively; P_TreatmentxCondition_= 0.734, 0.569, and 0.384, respectively). Obese cats had greater LSTM (P_Condition_= 0.001). There was a trend for LSTM to change with treatment (P_Treatment_ = 0.066), but there was no effect of treatment x body condition interaction on LSTM (P_TreatmentxCondition_= 0.858).

### 3.3. Serum biochemistry and lipoprotein profile

Mean serum biochemistry and lipoprotein values fell within reference intervals published by the Animal Health Laboratory (Guelph, ON, Canada), and are presented in Table 4. Serum TAG, HDL-C and VLDL concentrations differed between lean and obese cats (P_Condition_= 0.001, 0.041, and 0.001, respectively). Concentrations of serum TAG and VLDL were greater in obese cats across all three treatments. Conversely, serum HDL-C was lower in obese cats, as compared to lean cats. Serum HDL-C concentrations were also affected by treatment (P_Treatment_ = 0.005); concentrations were higher with choline supplementation, as compared to control and L-carnitine. Serum CHOL and LDL-C concentrations were also greater with choline supplementation in all cats (P_Treatment_ = 0.005, and 0.042, respectively). However, neither CHOL or LDL-C were affected by body condition (P_Condition_ = 0.104, and 0.453, respectively). Concentrations of serum TAG and VLDL were not affected by treatment alone (P_Treatment_ = 0.340, and 0.340, respectively), but tended to increase with L-carnitine supplementation in obese cats (P_TreatmentxCondition_ = 0.064, and 0.064, respectively). There was also a trend for treatment to increase serum NEFA (P_Treatment_ = 0.099). However, neither body condition or treatment x body condition interaction affected NEFA (P_Condition_= 0.241, and P_TreatmentxCondition_ = 0.884). No significant treatment x body condition interactions were noted for CHOL, HDL-C and LDL-C (P_TreatmentxCondition_ = 0.619, 0.242 and 0.586, respectively).

**Table 4:** Fasted serum biochemistry, lipid and lipoprotein profile values of lean (*n* = 9) and obese (*n* = 9 for control and L-carnitine; *n* = 8 for choline) adult cats receiving supplemental dietary choline (378 mg/kg BW^0.67^), supplemental L-carnitine (200 mg/kg BW), or control (no additional supplement top-dressed onto a commercial extruded adult feline diet) and fed to maintenance energy requirements in a 3 x 3 Latin square design for 6-week periods.

**Analyte**	**Units**	**Body condition (BC)**	**Treatment (T)**			***P*** **-values**		
			**Control (*****n** =* **18)**	**Choline (*****n** =* **17)**	**L-carnitine (*****n** =* **18)**	**BC**	**T**	**BC X T**
TAG	mmol/L	Lean	0.22 ± 0.45	0.27 ± 0.45	0.23 ± 0.4	**0.001**	0.340	0.064
		Obese	0.43 ± 0.45	0.41 ± 0.45	0.49 ± 0.45			
CHOL	mmol/L	Lean	5.91 ± 0.34	6.64 ± 0.34	6.01 ± 0.3	0.104	**0.005**	0.619
		Obese	5.45 ± 0.34	5.90 ± 0.34	5.16 ± 0.34			
HDL-C	mmol/L	Lean	4.80 ± 0.28	5.43 ± 0.28	5.03 ± 0.2	**0.041**	**0.005**	0.242
		Obese	4.38 ± 0.28	4.65 ± 0.28	4.37 ± 0.28			
LDL-C	mmol/L	Lean	1.01 ± 0.19	1.09 ± 0.19	0.87 ± 0.1	0.453	**0.042**	0.586
		Obese	0.87 ± 0.19	1.07 ± 0.19	0.57 ± 0.19			
VLDL	mmol/L	Lean	0.044 ± 0.01	0.053 ± 0.01	0.047 ± 0.0	**0.001**	0.340	0.064
		Obese	0.087 ± 0.01	0.081 ± 0.01	0.098 ± 0.01			
NEFA	mmol/L	Lean	0.31 ± 0.06	0.37 ± 0.06	0.44 ± 0.0	0.241	0.099	0.884
		Obese	0.40 ± 0.06	0.40 ± 0.06	0.49 ± 0.06			
ALP	U/L	Lean	14.56 ± 1.44	14.33 ± 1.44	14.67 ± 1.4	0.067	**0.042**	0.123
		Obese	19.00 ± 1.44	16.51 ± 1.44	18.78 ± 1.44			
ALT	U/L	Lean	40.44 ± 3.22^Ad^	54.44 ± 3.22^Ac^	43.33 ± 3.22^A^	0.180	**0.012**	**0.002**
		Obese	39.44 ± 3.22^Ac^	38.99 ± 3.22^Bc^	43.11± 3.22^Ac^			
CREAT	mmol/L	Lean	116.78 ± 6.83	115.78 ± 6.83	108.22 ± 6.8	0.701	**0.010**	0.103
		Obese	116.89 ± 6.83	118.90 ± 6.83	116.00 ± 6.83			
GLUC	mmol/L	Lean	5.56 ± 0.42	5.54 ± 0.42	5.70 ± 0.4	0.058	0.476	0.827
		Obese	6.48 ± 0.42	6.34 ± 0.42	6.83 ± 0.42			
BUN	mmol/L	Lean	7.58 ± 0.33	7.33 ± 0.33	7.19 ± 0.3	0.213	0.282	0.247
		Obese	7.97 ± 0.33	7.85 ± 0.33	8.00 ± 0.33			

Values expressed as LSM ± SEM. Down a column, different upper case letter superscripts (A, B), represent significant difference between body conditions within a treatment, where a *p*-value of less than 0.05 is considered significant (represented in bold); Across a row, different lower case letter superscripts (c, d), represent significant difference between treatments within a body condition, where a *p*-value of less than 0.05 is considered significant; Repeated measures ANOVA with Tukey *post-hoc* test. TAG, triglycerides; CHOL, cholesterol; HDL-C, high-density lipoprotein cholesterol; LDL-C, low-density lipoprotein cholesterol; VLDL, very low-density lipoprotein cholesterol; NEFA, non-esterified fatty acids; ALP, alkaline phosphatase; ALT, alanine aminotransferase; CREAT, creatinine; GLUC, glucose; BUN, blood urea nitrogen; BW, body weight; BC, effect of body condition; T, effect of treatment; BC X T, effect of body condition by treatment interaction.

Treatment did affect serum ALP, ALT, and CREAT (P_Treatment_ = 0.042, 0.012, and 0.010, respectively). There were no differences between ALP means following a Tukey's *post-hoc*. Serum ALT concentrations were lower with control, as compared to the choline treatment. Conversely, L-carnitine supplementation reduced serum CREAT, as compared to both control and choline, in both lean and obese cats. Lean cats consuming choline had greater concentrations of serum ALT as compared to lean cats consuming control and L-carnitine, and obese cats consuming L-carnitine (P_TreatmentxCondition_ = 0.002). There was a trend for body condition to change serum GLUC (P_Condition_= 0.058). However, GLUC did not change with treatment or treatment x body condition interaction (P_Treatment_ = 0.476, and P_TreatmentxCondition_ = 0.827). Serum BUN was not affected by treatment, body condition, or treatment x body condition interaction (P_Treatment_ = 0.282, P_Condition_= 0.213, P_TreatmentxCondition_ = 0.247).

### 3.4. Indirect calorimetry

There was a trend for fasted EE to change with treatment (P_Treatment_ = 0.087; Table 5), but there was no change with body condition or treatment x body condition interaction (P_Condition_= 0.685 and P_TreatmentxCondition_ = 0.189). These outcomes did not change when fasted EE was adjusted for BW (P_Treatment_ = 0.088, P_Condition_= 0.756, and P_TreatmentxCondition_ = 0.191). Fed EE was not affected by treatment or treatment x body condition interaction (P_Treatment_ = 0.915, and P_TreatmentxCondition_ = 0.874), but tended to be lower in the obese cats (P_Condition_= 0.090). Treatment, condition and treatment x body condition interaction did not change fed EE when adjusted for BW or food intake (P_Treatment_ = 0.905, and 0.761, respectively; P_Condition_= 0.264, and 0.090, respectively; P_TreatmentxCondition_ = 0.881, and 0.767, respectively).

**Table 5:** EE and RQ of lean (*n* = 9) and obese (*n* = 9 for control and L-carnitine; *n* = 8 for choline) adult cats receiving supplemental dietary choline (378 mg/kg BW^0.67^), supplemental L-carnitine (200 mg/kg BW), or control (no additional supplement top-dressed onto a commercial extruded adult feline diet) and fed to maintenance energy requirements in a 3 x 3 Latin square design for 6-week periods.

	**Body condition (BC)**	**Treatment (T)**			***P*** **-values**		
		**Control (*****n** =* **18)**	**Choline (*****n** =* **17)**	**L-carnitine (*****n** =* **18)**	**BC**	**T**	**BC X T**
EE fasted, kcal/kg BW/day	Lean	34.06 ± 2.91	32.96 ± 2.90	39.31 ± 2.9	0.685	0.088	0.189
	Obese	32.90 ± 2.86	34.63 ± 2.92	34.46 ± 2.86			
Adjusted for BW^$^	Lean	32.11 ± 4.57	31.02 ± 4.54	37.29 ± 4.6	0.756	0.088	0.191
	Obese	34.73 ± 4.37	36.62 ± 4.63	36.38 ± 4.49			
EE fed, kcal/kg BW/day	Lean	37.73 ± 2.90	35.53 ± 2.70	37.37 ± 2.8	0.090	0.915	0.874
	Obese	33.82 ± 2.42	33.76 ± 2.56	32.88 ± 2.35			
Adjusted for food intake^#^	Lean	35.49 ± 2.21	35.74 ± 2.31	38.24 ± 2.4	0.090	0.761	0.767
	Obese	33.19 ± 2.02	32.68 ± 2.10	33.01 ± 2.02			
Adjusted for BW^$^	Lean	39.07 ± 3.82	36.76 ± 3.69	38.77 ± 4.0	0.264	0.905	0.881
	Obese	32.92 ± 3.11	32.72 ± 3.40	31.96 ± 3.08			
RQ fasted	Lean	0.78 ± 0.01	0.79 ± 0.01	0.78 ± 0.0	0.905	0.497	0.807
	Obese	0.78 ± 0.01	0.78 ± 0.01	0.79 ± 0.01			
Adjusted for BW^$^	Lean	0.81 ± 0.01	0.82 ± 0.01	0.80 ± 0.0	**0.009**	0.587	0.712
	Obese	0.76 ± 0.01	0.75 ± 0.01	0.75 ± 0.01			
RQ fed	Lean	0.81 ± 0.01	0.80 ± 0.01	0.81 ± 0.0	0.080	0.427	0.964
	Obese	0.80 ± 0.01	0.79 ± 0.01	0.79 ± 0.01			
Adjusted for food intake^#^	Lean	0.81 ± 0.01	0.81 ± 0.01	0.81 ± 0.0	0.057	0.729	0.741
	Obese	0.80 ± 0.01	0.79 ± 0.01	0.79 ± 0.01			
Adjusted for BW^$^	Lean	0.83 ± 0.01	0.82 ± 0.01	0.82 ± 0.0	0.060	0.354	0.958
	Obese	0.79 ± 0.01	0.78 ± 0.01	0.78 ± 0.01			
AUC_RQ_ fasted, RQ^*^min	Lean	53.88 ± 3.51	56.24 ± 3.51	57.89 ± 3.6	0.894	0.538	0.754
	Obese	55.71 ± 3.51	54.33 ± 3.62	57.12 ± 3.51			
AUC_RQ_ 0–120, RQ^*^min	Lean	89.96 ± 1.21	92.18 ± 1.25	90.91 ± 1.2	0.454	0.055	0.692
	Obese	90.08 ± 1.21	91.79 ± 1.25	89.64 ± 1.21			
AUC_RQ_ 120–480, RQ^*^min	Lean	283.96 ± 5.91	289.05 ± 6.03	291.31 ± 6.0	0.914	0.676	0.341
	Obese	289.77 ± 5.91	287.41 ± 6.03	288.14 ± 5.91			
AUC_RQ_ 480–820, RQ^*^min	Lean	283.97 ± 4.77	278.88 ± 4.87	281.42 ± 4.8	0.166	0.286	0.940
	Obese	279.88 ± 4.77	276.28 ± 4.87	276.99 ± 4.77			
AUC_RQ_ 820–1,300, RQ^*^min	Lean	370.39 ± 14.61	376.65 ± 14.82	378.22 ± 14.8	0.292	0.739	0.694
	Obese	367.68 ± 14.61	371.79 ± 14.81	364.93 ± 14.61			

Values expressed as LSM ± SEM; *P* < 0.05 (represented in bold), Repeated measures ANOVA with Tukey *post-hoc* test. EE, energy expenditure; RQ, respiratory quotient; BW, body weight; BC, effect of body condition; T, effect of treatment; BC X T, effect of body condition by treatment interaction.

# Individual food intake during calorimetry used as a covariate.

$ Individual BW at time of calorimetry used as a covariate.

Fasted RQ did not change with treatment, condition or treatment x body condition interaction (P_Treatment_ = 0.497, P_Condition_= 0.905, P_TreatmentxCondition_ = 0.807). When adjusted for BW, fasted RQ was lower in the obese cats (P_Condition_= 0.009). The effects of treatment and treatment x body condition interaction remained insignificant (P_Treatment_ = 0.587, and P_TreatmentxCondition_ = 0.712). Fed RQ was similarly not affected by treatment or treatment x body condition interaction (P_Treatment_ = 0.427, and P_TreatmentxCondition_ = 0.964), but there was a trend for fed RQ to change with body condition (P_Condition_= 0.080). These outcomes remained the same when fed RQ was adjusted for both BW (P_Treatment_ = 0.354, P_Condition_= 0.060, and P_TreatmentxCondition_ = 0.958), and food intake (P_Treatment_ = 0.729, P_Condition_= 0.057, and P_TreatmentxCondition_ = 0.741).

There was a tendency for AUC_RQ_ 0–120 min postprandial to change with treatment in both lean and obese cats (P_Treatment_ = 0.055). However, no effect of body condition or treatment x body condition interaction was observed (P_Condition_= 0.454, and P_TreatmentxCondition_ = 0.692). Fasted AUC, and the remaining postprandial AUC_RQ_ intervals (120–480, 480–820, and 1,300 min) were not affected by body condition (P_Condition_ = 0.894, 0.914, 0.166, and 0.292, respectively), treatment (P_Treatment_ = 0.538, 0.676, 0.286, and 0.739, respectively), or treatment x body condition interaction (P_TreatmentxCondition_ = 0.754, 0.341, 0.940, and 0.694, respectively).

## 4. Discussion

To the authors' knowledge, this is the first study to investigate the effects of dietary choline supplementation in comparison to L-carnitine supplementation in obese and lean cats fed to maintain BW. Specifically, we sought to investigate lipotropic changes, as determined by serum lipid and lipoprotein profile, EE, RQ, and body composition data, between the two supplements. Previously, we reported that dietary choline at 5 and 6 x NRC RA increased serum lipid and lipoprotein profiles in overweight and obese cats, likely representing increased mobilization of hepatic lipids into circulation (21, 22). However, to allow the pet food industry and veterinary clinicians to make informed decisions regarding dietary supplementation and feline weight loss, it is important to assess the lipotropic effects of choline at the previously determined dose of 6 x NRC RA against L-carnitine (22), a supplement that has been frequently researched and used by the pet food industry (26–28). The dose of L-carnitine supplemented to the cats in the present trial was similarly based on previous research in overweight and obese cats (26, 28, 47, 48).

As expected, obese cats had greater food intake, energy intake, BW, BCS, and body composition values (total tissue mass, BF %, fat mass and LSTM), when compared to lean cats. These obese cats were specifically enrolled in this study due to their increased adiposity and were fed at energy intakes to support their current BW and BCS. A limitation of the present study was the approach to defining adiposity at enrollment, as the cats were classified as obese based solely on BCS. Body composition was not assessed by DXA at enrollment to evaluate BF % of the cats before group allocation and treatment. Although BF % differed between the lean and obese groups, the assigned mean BCS of the obese cats was higher than the respective mean BF % analyzed by DXA. The mean BF % of the obese cats was 32%, which corresponds with BCS 7/9 (36). Overall, all cats in the obese group could still be considered overweight or obese based on BF % (36), and BF % was still greater than in the lean cats. Because BCS is a subjective method of assessment, the BCS of the cats may have been overestimated due to localized fat deposits and/or abdominal fat pads which may have consisted of skin only. Similar limitations in investigator bias have previously been published in feline research (49, 50). Furthermore, differences in DXA machine, protocol, and computer software used by researchers can lead to differences in body composition and fat mass measurements among feline obesity studies (51).

Obesity results in alterations in the secretion of adipokines, leading to the development of other secondary health concerns such as insulin resistance and dyslipidemia (52–54). The obese cats in the present study had differences in their serum lipid and lipoprotein profiles, independent of treatment, when compared to the lean cats. Specifically, obese cats had overall concentrations of TAG and VLDL that were 59% greater than the lean cats. Additionally, overall concentrations of serum HDL-C were 13% lower in the obese cats compared to lean cats. To the authors' knowledge, there are no published reference ranges in cats for serum TAG, VLDL or HDL-C. However, the concentrations of serum TAG, VLDL and HDL-C reported herein were similar to those previously reported in overweight and obese adult cats receiving supplemental choline (21, 22). Serum TAG concentrations were also similar to those previously reported in healthy adult cats (55, 56). The present results align with previous reports of similarly higher circulating TAG, VLDL and lower HDL-C in obese cats, as compared to a lean control group (57–60). However, said authors also reported greater circulating NEFA, CHOL, and LDL-C concentrations in obese cats, which was not observed in the present study. As the mean age of the cats in the present study was lower than the mean age reported in these previous studies (57, 60), it is unclear if age may have also had an impact. To our knowledge, the effect of age on serum lipid, biochemistry and/or metabolomic profiles in overweight or obese cats has not specifically been investigated. However, cats under the age of two were reported to have lower serum CHOL and LDL-C concentrations as compared to cats over the age of two (BCS unknown) (61). Additionally, as these cats gained weight during a 12-week modified *ad libitum* feeding protocol during growth (25), there is a question of whether acute vs. chronic obesity may also impact lipoprotein profile and circulating metabolites. It is also worth considering that the increase in serum LDL and CHOL with choline treatment in both the lean and obese cats in the present study may have masked an effect of body condition that may have otherwise been observed. The same is possible for serum NEFA, where L-carnitine had a tendency to increase concentrations across both lean and obese cats.

The choline treatment in the present study provided a daily dietary choline intake of 6 x NRC RA, whereas cats on the L-carnitine and control treatments consumed a daily average choline intake of 1.2 and 1.3 x NRC RA, respectively. Both lean and obese cats had greater concentrations of CHOL, HDL-C-and LDL-C with the choline treatment, as compared to L-carnitine treatment (11, 7 and 33 % greater, respectively) and control (9, 9 and 13% greater, respectively). Serum CHOL concentrations remained within reference range for all treatments (2–12 mmol/L), and serum HDL-C and LDL-C concentrations were similar to those previously reported in obese and overweight adult cats consuming supplemental choline (21, 22). The greater concentrations of CHOL, HDL-C and LDL-C with choline in the present study are not surprising as similar changes were previously observed in obese and overweight cats consuming choline at 5 and 6 x NRC RA daily, respectively (21, 22). Choline is a precursor for PC, which is considered essential in the formation of VLDL. The VLDL are responsible for packaging TAG and CHOL, and exporting them out of the liver and into circulation (16). Similarly, both HDL-C and LCL-C have important roles in CHOL transport and circulation throughout the body, and require PC for their assembly (62, 63). Nascent HDL-C also requires PC for its formation within the liver (64). Contrary to what was observed, it was expected that serum TAG and VLDL would increase with choline supplementation in the present study. The present findings are contrary to our previous findings in obese and overweight cats consuming choline at 5 and 6 x NRC RA, where both serum TAG and VLDL concentrations were greater as compared to control (21, 22). Instead, obese cats receiving the L-carnitine treatment tended to have the greatest concentrations of serum TAG and VLDL. These results contradict the current research that exists on L-carnitine supplementation in hyperlipidemic animals and obese human patients with non-alcoholic steatohepatitis, in which TAG concentrations were reduced or unchanged (65–69). Although Rahbar et al. (70) observed increases in fasting plasma TAG concentrations when L-carnitine was supplemented to human patients with type II diabetes mellitus, the number of cats enrolled in the current study and the differences in BF % between the lean and obese group may not have been sufficient to detect these differences.

Although fasted serum glucose concentrations remained within reference range (4.4–7.7 mmol/L) for all the cats, glucose tended to be 16% greater in the obese cats than in the lean cats in the present study. Impaired glucose tolerance and insulin resistance are common findings in obese cats (9, 71–73), and a 30% decrease in insulin sensitivity has been reported with each additional kg of BW in cats (73). Over time, the decrease in insulin sensitivity may result in the development of type II diabetes mellitus (74), which is considered four times more likely in obese cats, as compared to lean cats (75). It is unclear if the increased TAG concentrations in the obese cats receiving L-carnitine may have been reflective of decreased glucose tolerance. Similar results have not been published in cats, and the use of L-carnitine in the treatment of obesity and diabetes mellitus in cats requires further investigation.

Serum ALP concentrations in the present study were within reference range for all cats throughout all three treatment periods (12–60 U/L), but tended to be, on average, 22% higher in the obese cats when compared to the lean cats. To the author's knowledge, there have been no published reports of increased ALP concentrations in healthy obese cats. However, in humans, a significant positive linear relationship between ALP and body mass index has been reported (76); where obese subjects had ALP concentrations that were on average 15 and 16% greater when compared to the non-obese and lean subjects, respectively (76, 77). This is because adipose tissue can express and release ALP into circulation (77). In the present study, it is likely that the greater serum ALP concentrations in the obese cats were representative of their increased adiposity, instead of their liver health. None of the cats showed any clinical signs of hepatic malfunction at any point throughout the study. However, serum liver enzyme values alone cannot adequately assess feline hepatic health and/or function. In the future, ultrasonography and histopathology of liver biopsies should be considered, along with pre- and postprandial bile acid profiles.

Conversely, lean cats consuming choline had the greatest concentrations of serum ALT. However, the concentrations similarly remained within reference range (31–105 U/L), suggesting that hepatobiliary diseases were absent in the present cats (78). Serum ALT concentrations were 26% greater in lean cats receiving the choline treatment as compared to control, and 20% greater as compared to the L-carnitine supplement. The present finding is unexpected, as ALT has been reported to increase in cases of non-alcoholic steatohepatitis resulting from choline deficiency in humans and in rodent models (79, 80). Additionally, these results contradict previous work by Verbrugghe et al. (81), where obese cats had higher ALT concentrations as compared to lean cats. Previously, choline supplementation did not affect serum ALT levels in overweight or obese cats (21, 22). The cats in the present study did not show signs of hepatic health concerns, regardless of treatment or body condition. However, as mentioned, hepatic health was not thoroughly investigated in the present study.

L-carnitine supplementation resulted in lower serum CREAT concentrations, as compared to the other two dietary treatments. This finding aligns with previous research in rodent models, where L-carnitine levels were inversely correlated with serum CREAT and renal function was improved with supplementation (82). The protective effects of L-carnitine on renal function are believed to be a result of its ability to reduce lipid peroxidation and free radical generation (83). Similarly, adult cats with chronic kidney disease (International Renal Interest Society Stages 1–2) consuming an average of 211 mg L-carnitine/kg BW from a test diet had serum CREAT levels that increased at a slower rate, as compared to the control group consuming an average of 2 mg L-carnitine/kg BW. However, the test diet and control diet differed in their overall nutrient profiles, and therefore the change in serum CREAT cannot be attributed to solely dietary L-carnitine intake (84). All cats in the present study were fed the same base diet, deemed in good health prior to enrollment with a complete blood count and serum biochemistry profile, and showed no signs of renal health concerns throughout the trial. Urinalysis and symmetric dimethyl arginine concentrations were not evaluated but should be considered for future L-carnitine studies.

Food and energy intakes were 0.3 % lower with choline treatment when compared to L-carnitine treatment, and 3% lower when compared to control. It is unclear whether the differences in food intake between treatments may have affected circulating metabolite concentrations, and to what effect. However, this reduction was not enough to influence BW, BCS, BF % and/or fat mass over the treatment periods. Similarly, it is also unlikely that this level of energy reduction was enough to put the cats in a negative energy balance and cause the observed lipid mobilization with choline treatment. It is unclear whether the change in food intake was due to palatability or due to a potential effect of satiety. A similar reduction in food intake was previously reported in growing kittens consuming choline at 3 x NRC RA. However, there were no changes in the analyzed fasted serum satiety hormone concentrations (ghrelin, leptin, glucagon-like peptide-1, peptide-YY or gastric inhibitory polypeptide) of said kittens with choline treatment (25). The mechanism through which choline may influence food intake in cats requires further elucidation.

Although there were no differences between dietary treatments on FM or BF %, there was a trend for greater LSTM in both obese and lean cats with treatment. On average, LSTM was 1.5% greater with choline treatment as compared to control, and 0.8% greater as compared to L-carnitine treatment. Supplementing betaine (a derivative of choline) in livestock species such as pigs has resulted in increased LSTM (23, 24). Although the exact mechanism has not been identified, it is presumably due to the role that betaine has as a possible methyl donor for the re-methylation of methionine; thus sparing methionine for protein synthesis and leading to increased s-adenosylmethionine production (85). There were no differences in LSTM with choline supplementation at 3 x NRC RA in growing kittens being fed *ad libitum* (25). However, supplemented kittens had lower food and subsequent protein intakes which may have impacted these results. In the present study, LSTM was 0.8% greater with L-carnitine compared to control. This observation aligns with previous research by Center et al. (26) in which overweight cats consumed a weight loss diet supplemented with 0 (control), 50, 100, or 150 mg carnitine/kg diet. Cats supplemented with carnitine achieved an increase in LSTM and a decrease in FM sooner (by day 42) compared to the cats receiving control (by day 84). These differences were attributed to lower RQ with treatment, indicating increased fat oxidation. Similarly, underweight and lean cats (BCS: 2.5–4/5) fed at 120% MER had lower fat deposition when supplemented 188 mg L-carnitine/kg diet compared to 121 mg L-carnitine/kg diet (28). However, the mechanism of action was unclear as there were no changes in EE, RQ or voluntary physical activity with dose throughout said 16-week trial.

Although EE is dependent on LSTM (86), the differences in LSTM between choline and the remaining treatments in the present study were minor and were likely not enough to elicit changes in EE. Instead, choline tended to increase RQ in the 120-min postprandial period, as compared to the other two treatments. The increase in RQ immediately post-feeding (0–120 min) may suggest a greater reliance on carbohydrate oxidation and less on gluconeogenesis with choline supplementation. This finding was not previously observed in overweight and obese cats or in guinea pigs supplemented with choline (21, 22, 87). It remains unclear why this may have occurred in the present study. There was a trend for greater fasted EE across all cats with the L-carnitine treatment. These findings align with previous research investigating L-carnitine supplementation in overweight cats. Overweight cats receiving 100 mg L-carnitine/kg BW similarly had greater fasted EE after 21 and 42 days of supplementation, as compared to overweight cats fed control (27). Although fed EE did not change with treatment in the present study, there was a trend for lower fed EE and fed RQ in the obese cats, as compared to the lean cats. Lower EE in overweight and obese cats as compared to lean cats is a common finding as an animal's surface area will influence EE (27, 86, 88). Perseghin et al. (89) similarly observed that overweight human participants had lower fed RQ when compared to lean participants, although not significant. The authors suggested that the increased leptin in the overweight participants may have played a role in promoting fatty acids toward oxidation instead of storage (90). However, said overweight study participants had normal insulin and leptin sensitivity. Although there are reports of decreased insulin and leptin sensitivity with weight gain in cats (71, 91), neither leptin nor insulin were measured in the obese cats in the present study and we therefore cannot conclude if the same association was present.

In agreement with previous publications, the present study found clear differences in circulating lipid and lipoprotein concentrations of obese and lean cats. Specifically, obese cats had greater concentrations of circulating TAG and VLDL, and lower concentrations of circulating HDL-C. Despite the differences associated with obesity, choline supplementation at six times the published RA resulted in greater HDL-C, CHOL and LDL-C in all cats. These findings are in alignment with our previous research in overweight and obese cats, which similarly found greater circulating lipid and lipoprotein concentrations with choline supplementation. These results suggest that choline supplementation may be beneficial in mobilizing lipids out of the liver and into circulation. Based on the findings of the present research, choline shows more promise than L-carnitine as a supplement for combating the risk of FHL in cats. While choline decreased food intake and had a tendency to improve LSTM, it did not increase EE in the same manner that L-carnitine did. Thus, future research should focus on evaluating the synergistic benefits of supplementing dietary choline and L-carnitine together to obese cats on a weight loss program. Together, L-carnitine and choline may prove useful in promoting weight loss, while maintaining LSTM and minimizing hepatic lipid stores in these cats.

## Data availability statement

The raw data supporting the conclusions of this article will be made available by the authors, without undue reservation.

## Ethics statement

The animal study was reviewed and approved by University of Guelph Animal Care Committee (AUP#4496).

## Author contributions

AV, MB, and GK secured funding for this research. AR, AS, MB, GK, and AV were responsible for designing this study. AR, SV-S, and AV were responsible for data collection. AR was responsible for statistical analysis and writing of the manuscript. All authors contributed to editing and reviewing this manuscript.
